# Characterization of the Oxidative Stress in Renal Ischemia/Reperfusion-Induced Cardiorenal Syndrome Type 3

**DOI:** 10.1155/2020/1605358

**Published:** 2020-10-09

**Authors:** Wellington Caio-Silva, Danielle da Silva Dias, Carolina Victoria Cruz Junho, Karine Panico, Raquel Silva Neres-Santos, Milena Trevisan Pelegrino, Joana Claudio Pieretti, Amedea Barozzi Seabra, Kátia De Angelis, Marcela Sorelli Carneiro-Ramos

**Affiliations:** ^1^Center of Natural and Human Sciences (CCNH), Federal University of ABC, Avenida dos Estados, 5001, 09210-170 Santo André, SP, Brazil; ^2^Department of Physiology, Federal University of São Paulo, Santo André 862, 04023-062 São Paulo, SP, Brazil

## Abstract

In kidney disease (KD), several factors released into the bloodstream can induce a series of changes in the heart, leading to a wide variety of clinical situations called cardiorenal syndrome (CRS). Reactive oxygen species (ROS) play an important role in the signaling and progression of systemic inflammatory conditions, as observed in KD. The aim of the present study was to characterize the redox balance in renal ischemia/reperfusion-induced cardiac remodeling. C57BL/6 male mice were subjected to occlusion of the left renal pedicle, unilateral, for 60 min, followed by reperfusion for 8 and 15 days, respectively. The following redox balance components were evaluated: catalase (CAT), superoxide dismutase (SOD), total antioxidant capacity (FRAP), NADPH oxidase (NOX), nitric oxide synthase (NOS), hydrogen peroxide (H_2_O_2_), and the tissue bioavailability of nitric oxide (NO) such as S-nitrosothiol (RSNO) and nitrite (NO_2_^−^). The results indicated a process of renoprotection in both kidneys, indicated by the reduction of cellular damage and some oxidant agents. We also observed an increase in the activity of antioxidant enzymes, such as SOD, and an increase in NO bioavailability. In the heart, we noticed an increase in the activity of NOX and NOS, together with increased cell damage on day 8, followed by a reduction in protein damage on day 15. The present study concludes that the kidneys and heart undergo distinct processes of damage and repair at the analyzed times, since the heart is a secondary target of ischemic kidney injury. These results are important for a better understanding of the cellular mechanisms involved in CRS.

## 1. Introduction

Cardiorenal syndrome (CRS) is the term used to define the pathological crosstalk between the heart and kidney through systemic inflammation. This syndrome has been described in five classical types: cardiorenal (types 1 and 2), renocardiac (types 3 and 4), and systemic (type 5) [1]. Specifically, CRS type 3 is described as an acute kidney injury (AKI) leading to heart failure. A common risk factor for the development of CRS type 3 is the renal ischemia/reperfusion (I/R) injury, and it is characterized by a rapid decrease in kidney function [1].

This process of I/R in the kidney induces a systemic inflammatory cascade that leads to an increase in the expression of inflammatory factors, such as interleukin 6 (IL-6) and tumor necrosis factor *α* (TNF-*α*), which aggravates renal injuries [2]. The high levels of these inflammatory factors are related to heart failure [3] and lead to cardiac hypertrophy via many pathways, such as nuclear factor kappa B (NF-*κ*B) activation [4, 5]. In addition to inflammation, an imbalance between antioxidants and oxidants is observed in both organs after I/R [6, 7]. This imbalance in favor of oxidant agents causes the accumulation of some reactive oxygen species (ROS) that can promote cellular injury. This process is defined as oxidative stress and plays an important role in several cellular signaling pathways by damaging proteins, lipids, and DNA leading ultimately to cell apoptosis [8, 9].

Many enzymes act to prevent and/or suppress the formation of ROS. Enzymes such as catalase (CAT) and superoxide dismutase (SOD) lead to the dismutation of the superoxide anion (O_2_^∙−^) and subsequent breakdown of hydrogen peroxide (H_2_O_2_) and hydroperoxides into water, alcohol, and oxygen [9]. It has been found that SOD plays a key role in the regulation and protection of kidney injury and renal blood flow hours after I/R [10]. On the other hand, the mechanisms to produce several ROS that are needed in basal level function as antioxidant counterbalance. The O_2_^∙−^, for example, is catalyzed by the enzyme nicotinamide adenine dinucleotide phosphate (NADPH) oxidase (NOX) and can react with nitric oxide (NO) at a near-diffusion limited rate to form peroxynitrite (ONOO^−^), and it is known to mediate several aspects of renal I/R injury [10].

The result of ROS damage at various levels in the body is directly related to the numerous pathologies demonstrating the biological relevance of redox regulation [8, 11, 12]. Based on these studies and the importance of oxidative stress in several cardiac and renal pathologies, including CRS, our main objective was to characterize the pattern of some important oxidant and antioxidant agents using a murine model, in order to observe its impact on cardiac hypertrophy and to better understand the connection and dependence between the heart and kidney.

## 2. Materials and Methods

### 2.1. Animals and Experimental Protocol

Adult male C57BL/6J mice 6-8 weeks old (22-28 g) were obtained from the Animal Facility of Federal University of ABC (São Paulo, Brazil). The animals were kept in temperature-controlled rooms (24°C) with a 12/12 h light/dark cycle and had free access to standard mice chow and water. All surgical procedures and protocols were approved by the Ethics Committee on Animal Use of the Federal University of ABC (no. 5231210417) in accordance with the National Council for Control of Animal Experimentation (CONCEA).

The protocol used to induce renal I/R was the same as that used by Trentin-Sonoda et al. [5, 13]. To reflect this, the animals were divided into three groups: control (sham) and renal ischemia followed by 8 and 15 days of reperfusion (I/R 8 d and I/R 15 d, respectively). Briefly, the I/R mice were anesthetized and opened with an abdominal incision, and the left renal pedicle was occluded with a steel clamp for 60 min. In the case of the sham group, only an abdominal incision was performed without pedicle occlusion. The kidneys and heart were weighed and stored at -80°C, and the blood was collected by puncturing the inferior vena cava. The blood was centrifuged at 4°C, at 12000 × *g* for 15 min, to obtain the serum or plasma (using EDTA).

### 2.2. Tissue Preparation

The tissue was homogenized in an Ultra-Turrax blender using 5 mL of tissue buffer (140 mM KCl, 20 mM sodium potassium buffer, pH 7.4) for 1 g of tissue. The homogenate was centrifuged at 600 × *g* for 10 min at 2°C. The protein concentration was determined using the Lowry protein assay [14], using bovine serum albumin (BSA) with a concentration of 1 mg/mL as a standard.

### 2.3. Oxidative Stress Analyses

Protein oxidation levels were measured as described by Reznick and Packer [15]. In this method, the protein carbonyl groups react with 2,4-dinitrophenylhydrazine (DNPH) to form 2,4-dinitrophenylhydrazone, which can be measured spectrophotometrically at 360 nm. Data were expressed as nmol/mg of protein extract.

Lipid oxidation levels were measured by the chemiluminescence method [16], in which a scintillation spectrometer (Tri-Crab 2800 TR, PerkinElmer) was utilized, using the tritium channel mode at room temperature. The sample was diluted in tissue buffer in scintillation vials; 3 mM tert-butyl hydroperoxide was added. Finally, chemiluminescence was determined by measuring the maximal level of emission. Calculations were made as described, based on a standard curve. Data were expressed as cps/mg of protein, with cps representing counts per second.

### 2.4. Oxidant Agent Analyses

NOX activity in the sample was determined by the production of superoxide, using 50 mM phosphate buffer containing 2 mM EDTA and 150 mM sucrose, 1.3 mM NADPH, and 10 *μ*L of sample. Superoxide production was expressed as nmol/min/mg protein [17].

S-Nitrosothiols and nitrite content were obtained using an amperometric method [18, 19]. Total tissue protein was extracted using RIPA lysis buffer, and the protein concentrations were determined using the BCA kit (Thermo Scientific). Aliquots of 100 *μ*L containing 1 *μ*g/*μ*L of protein were added to the vial containing 15 mL of an aqueous solution of copper chloride (0.1 mol L^−1^) for determination of total S-nitrosothiol (RSNO) content or to an aqueous solution of potassium iodide (0.1 mol L^−1^) in sulfuric acid (0.1 mol L^−1^) for determination of nitrite (NO_2_^−^) content. The NO generated from RSNO or from NO_2_^−^ in the samples was detected using the WPI TBR4100/1025 free radical analyzer (World Precision Instruments Inc., Sarasota, FL, USA), using a nitric oxide-specific ISO-NOP sensor (2 mm). The experiments were performed in triplicate, and the calibration curves were obtained with aqueous solutions of freshly prepared S-nitrosoglutathione (GSNO) for RSNO or with sodium nitrite (NaNO_2_) for NO_2_^−^. Data were compared to the standard curves obtained with GSNO or with NaNO_2_. The results of the samples were compared with the standard curve and normalized to pmol/g of protein.

Hydrogen peroxide tissue content was measured by the oxidation of phenol red, mediated by radish peroxidase (HRP), leading to the formation of an H_2_O_2_ compound measurable at 610 nm [20]. The results were expressed in *μ*M of H_2_O_2_/mg of protein.

### 2.5. Antioxidant Agent Analyses

SOD activity was determined by the inhibition of the reaction between O_2_^∙−^ and pyrogallol, as described by S. Marklund and G. Marklund [21]. The assay was measured spectrophotometrically by the rate inhibition of pyrogallol autooxidation at 420 nm. Enzyme activity was reported as U/mg protein (U being equivalent to the amount of enzyme that inhibits the oxidation rate of the detector by 50%).

CAT activity was determined by measuring the decrease in H_2_O_2_ concentration at 240 nm [22]. Absorbance was read in quartz cubes with 10 *μ*L of H_2_O_2_ at 0.3 M for 10, 20, 30, 40, 50, and 60 s. Calculations were made as described based on a standard curve. The results were reported as nmol of H_2_O_2_/mg protein.

Total antioxidant activity was determined using the ferric reducing/antioxidant power (FRAP) assay [23]. Sodium acetate and acetic acid buffer solution were mixed with a standard solution of ferrous sulfate heptahydrate or sample stored in microplates. The microplates were measured spectrophotometrically at 593 nm. The value was expressed in mM of Fe(II).

### 2.6. Gene Expression Analyses

Total RNA was extracted as described in the Trizol® datasheet. Briefly, 1 mL of Trizol® was used for each 100 mg of tissue and processed in Polytron (Kinematica). The samples were treated with chloroform, and isopropanol was used to precipitate the RNA. The pellet was then washed with 75% ethanol. They were diluted and stored in diethylpyrocarbonate- (DEPC-) treated water. RNA concentration was measured using a NanoDrop Lite spectrophotometer (Thermo Scientific©, San Jose, CA). After reverse transcription using the ProtoScript kit (New England BioLabs®), the final cDNA product was used for real-time PCR (qPCR) (Rotor-Gene Q, QIAGEN's real-time PCR cycler) to quantify gene expression. The primers were designed using Primer BLAST and BLAST NCBI. The 2^-*ΔΔ*CT^ method was used to calculate changes among the groups, with cyclophilin A as a housekeeping gene.  Table 1 shows the primer sequences used for qPCR amplification.

### 2.7. Statistical Analysis

The Levene test was used to evaluate data homogeneity. Analysis of variance (ANOVA) and the subsequent Bonferroni test were performed to compare the mean values of more than two experimental groups. All comparisons were considered significant at *P* < 0.05. Data are expressed as mean ± standard error (SEM).

## 3. Results

### 3.1. Renal Ischemia and Reperfusion Increased Oxidant Activity in the Heart

Initially, we induce a unilateral renal ischemia for 60 minutes with subsequent reperfusion as already established by our group [5]. It is known that this model can lead to renal injury once urea, creatinine, and vimentin levels are increased after the first day of reperfusion [5]. This renal injury has been observed to initiate an inflammatory signal cascade after 8 days of reperfusion that functioned in tandem with the cardiac hypertrophy displayed in this model and subsequently produced results that could be considered similar or close to CRS type 3 [5]. Given the above, the redox balance was analyzed to integrate our data body once it had already been linked with other models of CRS. Since our model of renal I/R leads to inflammation and inflammatory components can be triggered by ROS activation, the levels of oxidant agents were measured.

**Figure 1:**
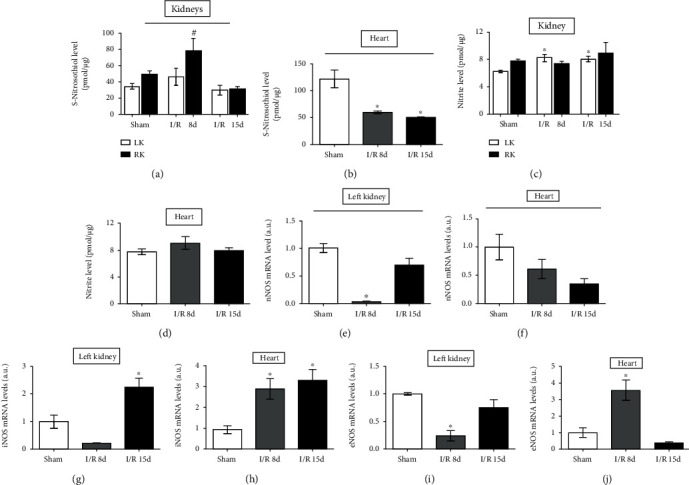
S-Nitrosothiol, nitrite dosages, and mRNA levels in heart and kidney tissues. NO bioavailability in renal tissue. (a) Total tissue S-nitrosothiol concentration in the kidneys (*n* = 5). (b) Total tissue S-nitrosothiol concentration in the heart (*n* = 4). (c) Total tissue nitrite concentration in the kidneys (*n* = 5). (d) Total tissue nitrite concentration in the heart (*n* = 5). (e) Analysis of the gene expression of nNOS in the left kidney. (f) Analysis of the gene expression of nNOS in the heart. (g) Analysis of the gene expression of iNOS in the left kidney. (h) Analysis of the gene expression of iNOS in the heart. (i) Analysis of the gene expression of eNOS in the left kidney. (j) Analysis of the gene expression of eNOS in the heart. (sham, *n* = 5; I/R 8 d, *n* = 5; and I/R 15 d, *n* = 5). All samples were evaluated by real-time PCR. Data are expressed as the mean ± SEM. One-way ANOVA followed by the Bonferroni posttest for the selected pairs and number of animals within the bars. ^∗^*P* < 0.05 with respect to the corresponding sham group.

Regarding the tissue bioavailability of NO in the form of RSNO and NO_2_^−^ (Figures 1(a)–1(d)), it was possible to observe an increase in RSNO levels in the right kidney (RK) 8 d after I/R injury, returning to the baseline level on the 15 d time point, while the left kidney (LK) remained unchanged in both days analyzed (Figure 1(a)). On the other hand, NO_2_^−^ levels remained unchanged in the RK, while in the LK, there was a significant increase in both days analyzed (Figure 1(c)). In parallel, it was possible to observe a significant reduction in the bioavailability of NO by RSNO in the heart tissue (Figure 1(b)), while the levels of NO_2_^−^ remained unchanged at the analyzed times (Figure 1(d)). Regarding the enzyme responsible for NO synthesis, the mRNA expression of the nitric oxide synthase (NOS) was analyzed and a modulation in the expression of each NOS isoform was observed (Figures 1(e)–1(j)). It was possible to observe that the constitutive enzyme isoforms (nNOS and eNOS) in the LK suffered a reduction in their expression 8 days after injury, when compared to sham, returning to baseline levels at 15 d (Figures 1(e) and 1(i)) while the inducible isoform (iNOS) suffered an increase at 15 d after reperfusion (Figure 1(g)). On the other hand, it was possible to observe an increase in the gene expression of the endothelial NOS (eNOS) isoform after 8 days, returning to the basal level 15 d after I/R injury in the heart (Figure 1(j)). The neuronal NOS (nNOS) isoform remained unchanged in both days analyzed (Figure 1(f)) while the expression of the iNOS isoform suffered a significant increase in both analyzed times, when compared to sham (Figure 1(h)).

**Figure 2:**
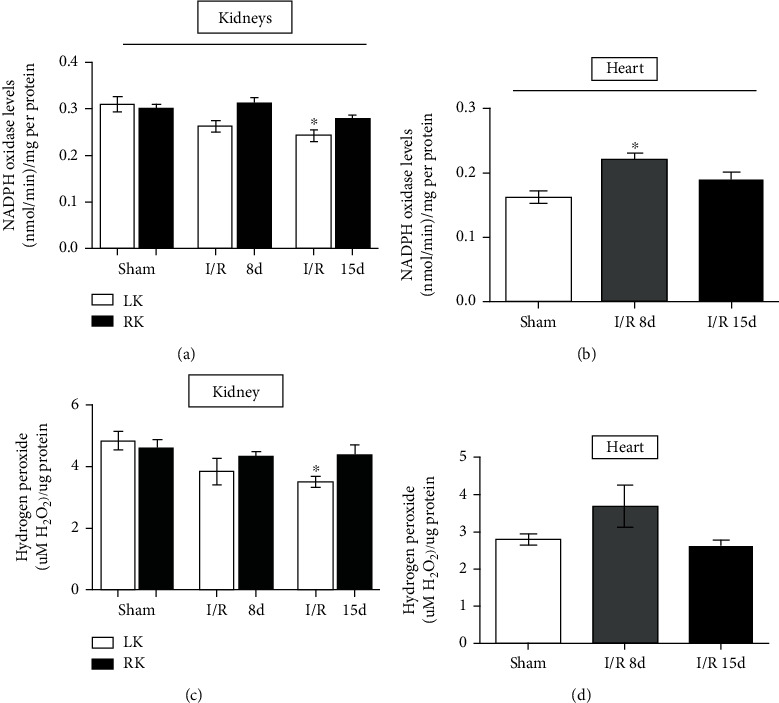
Oxidant agents in heart and kidney tissues. (a) NADPH oxidase activity in the kidneys. (b) NADPH oxidase activity in the heart. (c) Hydrogen peroxide levels in the kidneys. (d) Hydrogen peroxide levels in the heart. Heart assays (sham, *n* = 10; I/R 8 d, *n* = 6; and I/R 15 d, *n* = 5) and kidney assays (sham, *n* = 6; I/R 8 d, *n* = 6; and I/R 15 d, *n* = 7). Data are expressed as the mean ± SEM. One-way ANOVA followed by the Bonferroni posttest for the selected pairs and number of animals within the bars. ^∗^*P* < 0.05 with respect to the corresponding sham group.

We also measured the NOX enzyme activity and the H_2_O_2_ concentration levels in both tissues (Figure 2). Results showed that NOX activity in the LK was reduced after 15 days of reperfusion while the RK remains unchanged in both days analyzed (Figure 2(a)). Unlike the kidney, the NOX activity in the heart was increased after 8 days of reperfusion, returning to sham levels after 15 days (Figure 2(b)). The H_2_O_2_ levels in the kidneys were reduced in the LK 15 days after I/R, while the RK remains unchanged in both days analyzed (Figure 2(c)). In the heart, the H_2_O_2_ levels remained unchanged after I/R (Figure 2(d)).

These data indicate that there is an increase in the oxidation activity in the heart tissue at some levels, further highlighting that oxidative stress leaves the kidneys and reaches the heart tissue after reperfusion.

### 3.2. Oxidative Damage Is Reduced in the Kidney and Increased in the Heart in These Set Points

**Figure 3:**
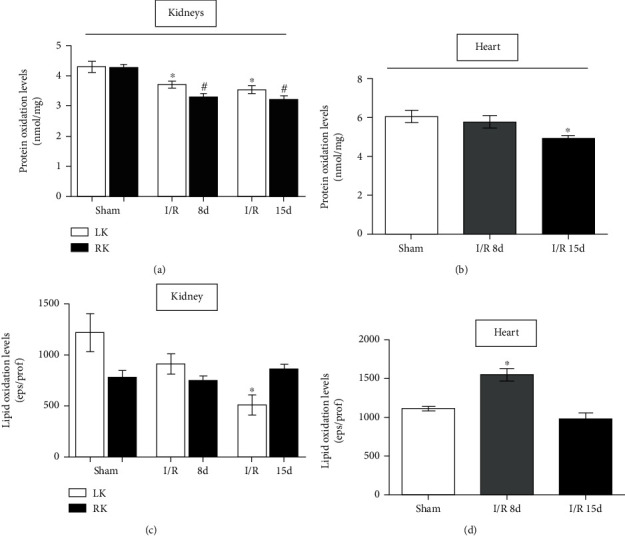
Oxidative stress by protein and lipid damage in the heart and kidney tissues. (a) Protein oxidation by carbonyl groups in the kidneys. (b) Protein oxidation by carbonyl groups in the heart. (c) Lipid peroxidation by chemiluminescence in the kidneys. (d) Lipid peroxidation by chemiluminescence in the heart. Heart assays (sham, *n* = 8; I/R 8 d, *n* = 6; and I/R 15 d, *n* = 7) and kidney assays (sham, *n* = 7; I/R 8 d, *n* = 6; and I/R 15 d, *n* = 6). Data are expressed as the mean ± SEM. One-way ANOVA followed by the Bonferroni posttest for the selected pairs and number of animals within the bars. ^∗^*P* < 0.05 with respect to the corresponding left kidney of the sham group. ^#^*P* < 0.05 with respect to the corresponding right kidney of the sham group.

ROS can damage important cellular compounds, such as proteins, lipids, and DNA [24]. Knowing that, we measured the protein and lipid oxidation levels in both heart and kidney tissues (Figure 3). The data shows a decrease in the oxidative damage in both protein and lipid content in the kidney (Figures 3(a) and 3(c)). In the heart, the lipid oxidation was increased after 8 days of reperfusion, when compared to the sham group (Figure 3(d)), while the protein oxidation levels were decreased after 15 days of reperfusion (Figure 3(b)). These results provide evidence that oxidative stress leaves the kidneys and reaches the heart tissue after reperfusion. This suggests that oxidative stress, besides contributing to kidney failure progression, is still capable of altering the morphology of the heart, promoting hypertrophy and the other damage cited previously.

### 3.3. Antioxidant Activity Only Occurs in the Kidney

**Figure 4:**
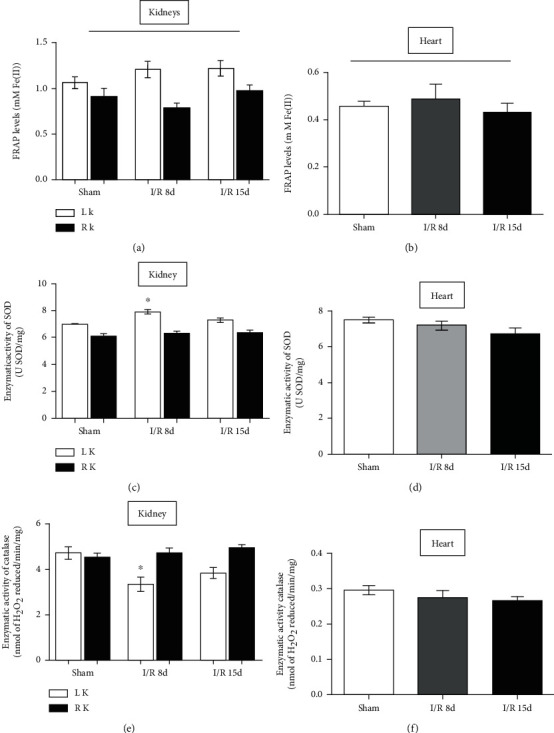
Antioxidant agents in heart and kidney tissues. (a) Total antioxidant activity by FRAP in the kidney. (b) Total antioxidant activity by FRAP in the heart. (c) Superoxide dismutase in the kidneys. (d) Superoxide dismutase in the heart. (e) Catalase activity in the kidneys. (f) Catalase activity in the heart. *N* = 6. Data are expressed as the mean ± SEM. One-way ANOVA followed by the Bonferroni posttest for the selected pairs and number of animals within the bars. ^∗^*P* < 0.05 with respect to the corresponding sham group.

We investigated the activity of some antioxidant agents in both the kidneys and heart tissues to see if there is a modulation as observed in the oxidant analyses (Figure 4). For that, the FRAP was used as a parameter to the total antioxidant activities in the tissue, while CAT and SOD were analyzed since they are the main enzymes responsible for protecting the cell and scavenging ROS [9, 23]. Results showed that the FRAP levels in both the kidneys and heart (Figures 4(a) and 4(b)) as well as SOD and CAT activity in the heart (Figures 4(d) and 4(f)) remained unchanged after I/R. On the other hand, the injured kidney (LK) showed a decrease in CAT activity and an increase in SOD activity 8 days after I/R, returning to sham levels after 15 days of I/R in both enzymes, while the RK remained unchanged (Figures 4(e) and 4(c)).

## 4. Discussion

In this study, we characterized some of the main components responsible for the redox balance during the cardiac hypertrophy model induced by renal I/R. These data suggest three main points: (i) the antioxidant activity is not modulated in the heart in these set points of reperfusion, leading to a tendency towards unbalanced oxidant/antioxidant, (ii) the antioxidant activity peak occurs prior to the 8^th^ day of reperfusion, and/or (iii) another antioxidant agents or compensatory mechanisms are being triggered once the damage is reduced even with the increase in some oxidant agents in both organs. Based on the analyses carried out so far, we were able to show a distinct modulation of the oxidant agents in each organ, the kidneys and heart. Together with subtle or unchanging antioxidant agent levels analyzed such as FRAP or CAT, a favorable imbalance could be allowed in the ROS production and damage, but in these analyzed time points, the damage was mostly reduced.

It is known that the pathogenesis of renal injury by I/R can be associated with NO deficiency in tissue [25]. The NO produced by the constitutive NOS isoform has several protective functions; however, NO from the iNOS has toxic effects in I/R [26–28]. Therefore, a decrease in NO derived from eNOS, and a consecutive increase in NO derived from iNOS in the kidneys, is associated with inflammation and vasoconstriction during the initial stage of reperfusion [26]. However, as we observed in this study, the increase in the expression of the iNOS isoform occurs only after 15 days; it is possible that this modulation occurs at different times of reperfusion, with an increase hours after ischemia in response to the injury as observed in these studies and another increase after few weeks in response to the increase in inflammatory cytokines at 8 d and organ atrophy as already observed by our group [5]. Once produced, NO can be rapidly converted mostly into NO_2_^−^, nitrate (NO_3_^−^), and RSNO working both as oxidant and antioxidant agents [29, 30]. Several studies have already demonstrated in I/R models the role of NO_2_^−^ as a potent mediator of cytoprotection by inhibiting mitochondrial complex I activity after ischemia, attenuating the generation of mitochondrial ROS. As the production of NO, during ischemia, is limited by the reduction in the expression of NOS, NO_2_^−^ probably works as a major source of NO generation independent of NOS in the organ [31]. Knowing that, we expected that the NO bioavailability was reduced in the kidney in our model by the consumption of this reservoir. It is possible that the modulation in the kidney in NO_2_^−^ and RSNO observed here is due to the previously increased activity in the NOS before these analyzed times.

Several studies have been linked with the NOX activity and H_2_O_2_ levels. It was found that NOX isoform 4 (NOX4) is the predominant isoform found in renal tissue and this isoform has a greater predisposition to generate H_2_O_2_ instead of anion superoxide [32, 33]. This isoform activity is elevated in cases of epithelial and tubular lesions playing a fundamental role in the oxidation process in the kidney [32]. Rajaram et al. [34] observed a reduction in the levels of NOX4 gene and protein expression, in the tubular region in parallel with the progression of fibrosis, in patients and animal models with chronic renal failure (CRF). The beta growth transforming factor (TGF-*β*) is one of the main regulators of NOX and has a fundamental role in the establishment of fibrosis and disease progression [35, 36]. Despite these studies, previous data from our laboratory indicate a reduction in TGF-*β* gene expression in 12 and 15 days after ischemia [37]. In this way, we can suppose that the general reduction observed in NOX is due to a reduction mainly in NOX4 as a consequence of the reduction in TGF-*β* observed in previous studies in our laboratory. The reduction of H_2_O_2_ is also justified by this factor, since its production occurs mainly by NOX4 in the kidneys.

The unchanged kidney tissue FRAP levels observed in the present study corroborate the results by Gyurászová et al. [38], which were demonstrated after comparing the total antioxidant capacity in the serum of Wistar rats with AKI (48 hours after injury) and CRF (6 months after injury). They also verified through the FRAP technique that the reducing power in the serum of animals with ARI remained unchanged, while in the CRF, the FRAP was increased in relation to the sham [38]. Possibly, there may be an occurring transition process between AKI and CRF at the times proposed in this study, so the absence of modulation in FRAP levels could represent the transition from a reduction in FRAP observed in very acute times (24 hours after injury) and the increase in FRAP levels observed in more chronic times (6 months after injury). Even though the SOD and CAT are reported to be reduced in I/R injury [6], Singh and collaborators [39] observed something similar to the one presented here where the mitochondrial SOD isoform is found to increase in the I/R-damaged kidney after 24 hours of reperfusion. Still in that study, Singh et al. also found that the intracellular increase in H_2_O_2_ during the I/R injury process leads to an inactivation of CAT by the formation of a complex between both “CAT” and “H_2_O_2_” [39]. So, it is possible that in more chronic postinjury times such as 8 d, this SOD isoform levels become more expressive leading to a significant increase in the general activity also explaining the CAT activity reduction and corroborating the H_2_O_2_ increase observed. Together with this, based on data already published by our group, at the fifth day after I/R, a peak is observed in the expression of TNF-*α* in this model [5]. It is already known that TNF-*α* can modulate the SOD, so it is possible to assume that the increase observed in the general SOD activity at 8 d is due to the stimulus induced by the peak of TNF-*α* on the fifth day [5, 40].

Most of the studies have shown the important role of the cell damage in the establishment of AKI and chronic kidney injury due to the excess of oxidizing agents and oxidative stress in the tissue [6, 41, 42]. However, based on the data presented here, we can observe a reduction in damage and in some oxidizing agents, weeks after the I/R injury. This period of apparent renoprotection may be due to a significant increase in the autophagy process. Some studies have already identified that in stress situations, such as that observed in AKI, the autophagy process is activated, capable of degrading and recycling damaged organelles and macromolecules in an attempt to maintain cellular homeostasis [43]. Kaushal and Shah [43] also observed that the formation of autophagosomes is reduced in acute times leading to the process of apoptosis. This corroborates other studies on a renal I/R model that identified a reduction in the levels of enzymatic activity of SOD and CAT 24 hours after I/R injury, with a consequent increase in cell damage [6]. However, the data presented here reveal that in more chronic times, more precisely 8 and 15 days after injury, there is a reduction of cell damage at lipid and protein levels.

In the heart, NO plays important physiological roles by regulating calcium levels, increasing the contraction of cardiomyocytes, reducing vascular tone, preventing atherosclerosis, and attenuating ventricular hypertrophy [44]. In pathological conditions, its function is still contradictory, since a worsening of hypertrophy and dysfunction caused by aorta constriction has already been observed, due to the reduction of bioavailable NO in eNOS-knockout mice; also, an improvement in hypertrophy due to the lack of uncoupled eNOS has also been seen in another similar experiment as mentioned before [45, 46]. Thus, the NOS modulation can have both protective and antiprotective roles. Studies have also shown the close relationship between the increase in angiotensin II and the increase in eNOS expression via a signaling cascade by angiotensin receptors [47]. Beside this increase, Amador-Martínez and his group [48] found that even with this eNOS increase, the heart tissue showed a decrease in eNOS enzyme activity. Based on these data, it is possible to assume that despite high expression on 8 d, eNOS is in a decoupled state, generating ROS such anion superoxide other than NO. This is in line with the observed reduction in RSNO levels in the tissue. When we analyzed the NOX activity, we observed a similar behavior as observed by other studies that have shown that the increase in the levels of angiotensin II and inflammatory cytokines is capable of inducing an increase in NOX activity and this in turn induces cardiac remodeling [49, 50]. This corroborates what was observed in our previous laboratory studies, where around the 5th and 8th days after injury, inflammatory cytokines such as TNF-*α*, interferon gamma (IFN-*γ*), and interleukin-1*β* (IL-1*β*) are being expressed at high levels in cardiac tissue [5]. Thus, it is possible that the increase in NOX is a response to the increase in inflammatory cytokines in the same period. It is interesting to note that the increase in NOX did not influence the levels of H_2_O_2_ in the tissue, since the superoxide anion produced by the enzyme is rapidly dismutated into H_2_O_2_. In this way, it is possible that there is an increase only in the levels of superoxide anion, and this increase, together with the NO, is being rapidly converted into ONOO^−^ thus inducing the lipid damage.

In cardiac hypertrophy processes, metabolic changes, such as suppression of fatty acid consumption and increase in glucose consumption, can lead to the accumulation of lipids in the heart [51]. This accumulation can lead to lipotoxicity, inducing lipoperoxidation and apoptosis, causing myocardial dysfunction [52]. These results corroborate our findings here and those of the other group [53], once the increase in lipid damage occurs at the same time as the increase in NOX activity. Borchi et al. [53] observed a relationship between the NOX increase and lipoperoxidation in cardiac cells exposed to I/R. The activation of kinases, such as extracellular signal-regulated kinase (ERK), and cell death programming were related to NOX activity, since the use of NOX inhibitors leads to the prevention of lipoperoxidation and ERK activation [53].

It is known that the antioxidant deregulation and oxidative stress in cardiac tissue are fundamental for the establishment of pathological cardiac hypertrophy [54, 55]. However, the CRS model used here has subtle cardiac remodeling when compared to other models of cardiac hypertrophy. In this way, the heart probably can use other compensation and protection mechanisms, since the organ is a secondary target in this syndrome. This can occur as observed in cases of remote ischemic preconditioning where an I/R process in peripheral organs and tissues induces a cardioprotective effect [56]. Although the mechanism behind this conditioning process is still poorly understood, it is known that ROS play an important role in the remote ischemic preconditioning process [56].

**Figure 5:**
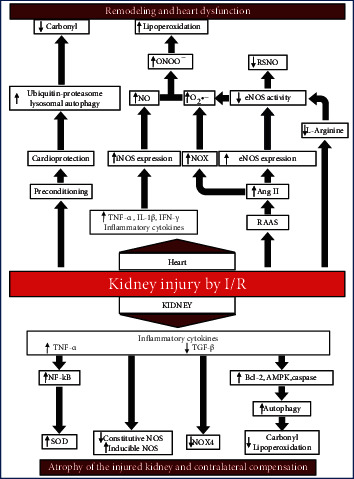
Flowchart of possible modulations in the model according to the bibliographic survey and data obtained here.

In conclusion, as summarized in Figure 5, according to these shown data and previous studies using this model, we proposed a possible mechanism that occurs in the time points of 8 and 15 days of reperfusion. The kidney injury goes through a renoprotection process, observed by the reduction of oxidant agents and reduction in the damage caused by oxidative stress, while the heart undergoes an increase in the levels of oxidant agents, leading to tissue damage at the lipid level. In addition, NO by-products such as RSNO and NO_2_^−^ probably have a protective character during the analyzed times.

## Figures and Tables

**Table 1:** List of primers.

Primers	Forward	Reverse
nNOS	TCGATGCCAAGGCTATGTCC	CCTTGTAGCTCTTCCTCTCCTC
iNOS	GCTCTAGTGAAGCAAAGCCC	GGATTCTGGAACATTCTGTGCT
eNOS	CCCAGCCTCTCCAGCAC	GCCCATCCTGCTGAGCC
Cyclophilin A	AGCATACAGGTCCTGGCATC	AGCTGTCCACAGTCGGAAAT

## Data Availability

The data used to support the findings of this study are available from the corresponding author upon request.
